# Study protocol for putting the ‘Person’ in the PiCTuRE: an exploratory sequential mixed methods-based design, exploring how precision medicine is implemented and experienced by people living with a primary tumour of the craniospinal axis

**DOI:** 10.1186/s12885-025-13795-9

**Published:** 2025-03-07

**Authors:** Gerard Mawhinney, Helen Higham, Simon Leedham, Olaf Ansorge

**Affiliations:** 1 Present Address: Nuffield Department of Clinical Neurosciences, University of Oxford, John Radcliffe Hospital, Headley Way, Level 6, West Wing, Headington, Oxford, OX3 9DU UK; 2 Nuffield Department of Experimental Medicine, University of Oxford, Oxford, UK

**Keywords:** Precision oncology, Patient engagement, Tissue donation, Craniospinal tumours

## Abstract

**Introduction:**

Primary tumours of the brain and spine are rare, heterogeneous, and frequently associated with significant morbidity and mortality. Advances in precision oncology and personalised medicine offer the potential to accelerate diagnosis, improve clinical outcomes, and yield critical insights into the molecular biology of these cancers of unmet need. Despite this, patient engagement in this area remains limited. Well-organised neuro-oncological biorepositories—those that are clinically integrated, fully consented, and derived from routine care—are limited and fragmented, which impedes progress. Therefore, it is crucial to examine the barriers to tissue donation and data integration within the NHS by analysing patients’ lived experiences. The PiCTuRE (Personalised Consent in Tissue donation for neuroscience Research, lived Experiences) study aims to develop a digital platform that provides customised, individualised, and interactive support to assist patients in their decision-making regarding tissue donation for research and participation in related clinical trials.

**Methods & Analysis:**

PiCTuRE is a multistage, mixed-methods, exploratory sequential investigation aimed at understanding the lived experiences of individuals donating tissue for research. It consists of three phases: Phase 1 involves an online survey to collect lived experience data, followed by semi-structured interviews to further explore individual perspectives. Thematic analysis will be performed to identify key themes. In Phase 2, patient-reported experience data will be gathered through co-design and statistically analysed to validate content for the development of the digital platform. Phase 3 will refine this intervention through iterative cycles of Phases 1 and 2, in collaboration with patients with lived experience of brain or spine tumours, to prepare it for integration into routine clinical practice.

**Ethics and Dissemination:**

Ethical approval has been obtained via the Medical Sciences Interdivisional Research Ethics Committee (MS IDREC), University of Oxford (R79248/RE001). Findings will be disseminated via podium presentations, public patient initiatives in partnership with charities, in peer-reviewed publications and via social media.

**Trial Registration Number:**

ISRCTN12601034.

**Supplementary Information:**

The online version contains supplementary material available at 10.1186/s12885-025-13795-9.

## Strengths & limitations of this study

**Strengths**.

• Provides valuable insights into the lived experiences of individuals diagnosed with rare primary tumours of the craniospinal axis.

• Explores potential barriers to tissue donation for research and participation in molecular data-driven clinical trials.

• Findings are likely to be applicable to other oncological or rare disease populations.

• Integrating mixed-methods, patient-involved data offers deeper insights into research engagement challenges and more effective solutions.

• Data is collected across the UK, offering a broad national perspective.

**Limitations**.

• The UK-based data may not fully reflect the diversity of global populations.

• The nature of the diagnosis may contribute to a reluctance among these patients to participate in the research.

**Supplementary Information:**

The online version contains supplementary material available at 10.1186/s12885-025-13795-9.

## Introduction

Primary brain and spinal tumours are individually rare, accounting for less than 3% and 1% of all cancer diagnoses in the UK, respectively [1, 2]. Rare cancers, by definition, have an incidence of fewer than 6 cases per 100,000 people [3]. Glioblastoma, the most common primary brain tumour in the UK, affects 3–4 people per 100,000 [1]. Primary spinal tumours, including those of bone and soft tissue (sarcomas), are even rarer, with fewer than 120 cases diagnosed annually in the UK [ [2, 4].

Together, these tumours impose a significant burden on patients, their families, and society. Diagnoses are often delayed, and there are limited integrated translational research programmes (spanning from biology to clinical trials) dedicated to addressing these conditions.

Patients are entitled to a timely and accurate diagnosis, which serves as the foundation for the implementation of novel “personalised” or “precision” medicine approaches, typically defined in biological terms. Equally important, however, is the consideration of patients’ perspectives and experiences when undergoing “personalised” treatment for these tumours. It is essential to understand their priorities, address their concerns, and ensure that treatment strategies align with their values and preferences [5–9].

### What is personalised/precision medicine??

Personalised medicine refers to an approach that focuses on the prevention, diagnosis, and treatment of diseases based on an individual’s unique molecular profile [9, 10]. In the context of oncology, personalised or precision medicine is particularly relevant for neurological cancers, which are rare and pose significant challenges in management [11–13]. Precision oncology often involves advanced molecular diagnostics, such as paired blood-tumour whole genome sequencing (WGS), followed by tailored treatments—either established or experimental—that target specific molecular pathways identified through WGS.

This approach is rapidly transforming cancer care across all oncological cohorts, enhancing the potential for more effective and targeted interventions.

### How is personalised complex oncology care evolving??

Historically, individuals undergoing complex oncology surgeries were seldom invited to participate in research related to their condition, with the decision to offer research opportunities often made solely by the treating clinician [14]. However, recent advancements in cancer care have shifted from a traditionally paternalistic approach to a more person-centred, personalised model, emphasising shared decision-making and patient involvement [15].

### Patient empowerment through digital healthcare

Empowering individuals diagnosed with rare cancers has been associated with improved treatment outcomes, as well as enhanced emotional and psychological well-being [6]. Innovative tools such as Dynamic Consent (DC) [16] and the Oxford Video Consent (OxVIC) [17] have demonstrated the value of effective, interactive communication in addressing patients’ clinical care needs and informing them about research opportunities. These approaches have been shown to enhance clinical outcomes, increase research participation, and promote overall well-being.

### Securing biological samples for Next-Generation diagnostics, translational research, and precision oncology trials

Biobanking refers to the systematic collection, processing, storage, and management of biological samples, such as blood, tissue, or other bodily fluids, alongside associated clinical and demographic data. These resources are invaluable for advancing next-generation diagnostics, conducting translational research, and designing clinical trials for precision oncology.

The success of biobanking initiatives relies heavily on the willingness of individuals to donate their biological tissue for research purposes. This altruistic contribution forms the backbone of advancements in precision medicine, enabling the discovery of novel biomarkers, the identification of molecular targets, and the development of personalised treatment strategies.

To ensure the sustainability and effectiveness of biobanking, it is crucial to foster trust and transparency between researchers and participants. This includes clear communication about the purpose of the research, the use of donated samples, and the safeguards in place to protect participants’ data and privacy. Patient-centric approaches, such as dynamic consent models, can further encourage participation and engagement, enhancing the impact of biobanking on the future of cancer care.

### Barriers to tissue donation for research

The inherent vulnerabilities experienced by cancer patients often present substantial obstacles to tissue donation for research purposes [18] Several factors contribute to this hesitation among individuals living with tumours. Trust issues frequently arise, with concerns about how donated samples will be used and whether personal data will be adequately protected [19, 20] Clinical time pressures also play a significant role, as limited consultation time often precludes thorough discussions about tissue donation, leaving patients’ questions and concerns unaddressed [21]. Additionally, psychological, and social barriers can deter participation. These include fear of the unknown, such as uncertainties regarding the implications or risks of donation [21] stress and anxiety stemming from the emotional toll of their diagnosis, which can overwhelm patients and reduce their willingness to consent [22]; and a loss of confidence in the healthcare system, resulting from a diminished sense of agency or trust due to their illness experience [23]. Collectively, these barriers highlight the need for tailored approaches to engage and support patients in the donation process.

While existing studies have explored cancer patients’ general attitudes toward biobanking [19, 23, 24], there remains a critical gap in understanding the lived experiences of individuals diagnosed with primary brain or spinal tumours and their specific perspectives on tissue donation.

We suggest that the primary obstacle to advancing translational neuroscience research is not a lack of willing participants but rather the absence of effective engagement strategies, tailored communication, and appropriately timed consent processes.

Addressing these gaps is essential to ensuring access to high value biological samples, which are often removed during routine NHS procedures but remain underutilised due to barriers in the consent process. By fostering better engagement and trust, the potential for transformative research in precision oncology can be fully realised.

**PiCTuRE-** is an exploratory sequential mixed-methods study [25]. Its primary aim is to optimise support for complex patient cohorts in their decision-making processes regarding tissue donation for research through development of a digital consent platform.

## Methods and analysis

### Objective

To design a digital intervention aimed at providing individuals with primary brain or spinal tumours comprehensive support to enhance their decision-making processes regarding tissue donation for research.

### Patient and public involvement

Has been used to inform this protocol. Individuals with lived experience of having a primary tumour of the craniospinal axis reviewed and provided feedback on the themes and the questions subsequently developed for the online survey. The survey’s content, language, and tone were tested with six participants prior to its electronic launch. Additionally, recruited participants will have the opportunity to contribute to the development of the digital consent tool through a co-design process.

The qualitative aspects of this protocol have been designed with reference to the Consolidated criteria for reporting qualitative research (COREQ) checklist for interviews and focus groups [26].

### General design

PiCTuRE is a multistage, online, exploratory sequential mixed-methods study [25] aimed at examining the lived experiences of individuals with primary craniospinal tumours who are considering or have donated tissue for research. This design aims to address gap in current published literature by integrating qualitative and quantitative data to explore the complex behaviours within the oncology setting, providing a robust foundation for outcomes.

**Fig. 1:**
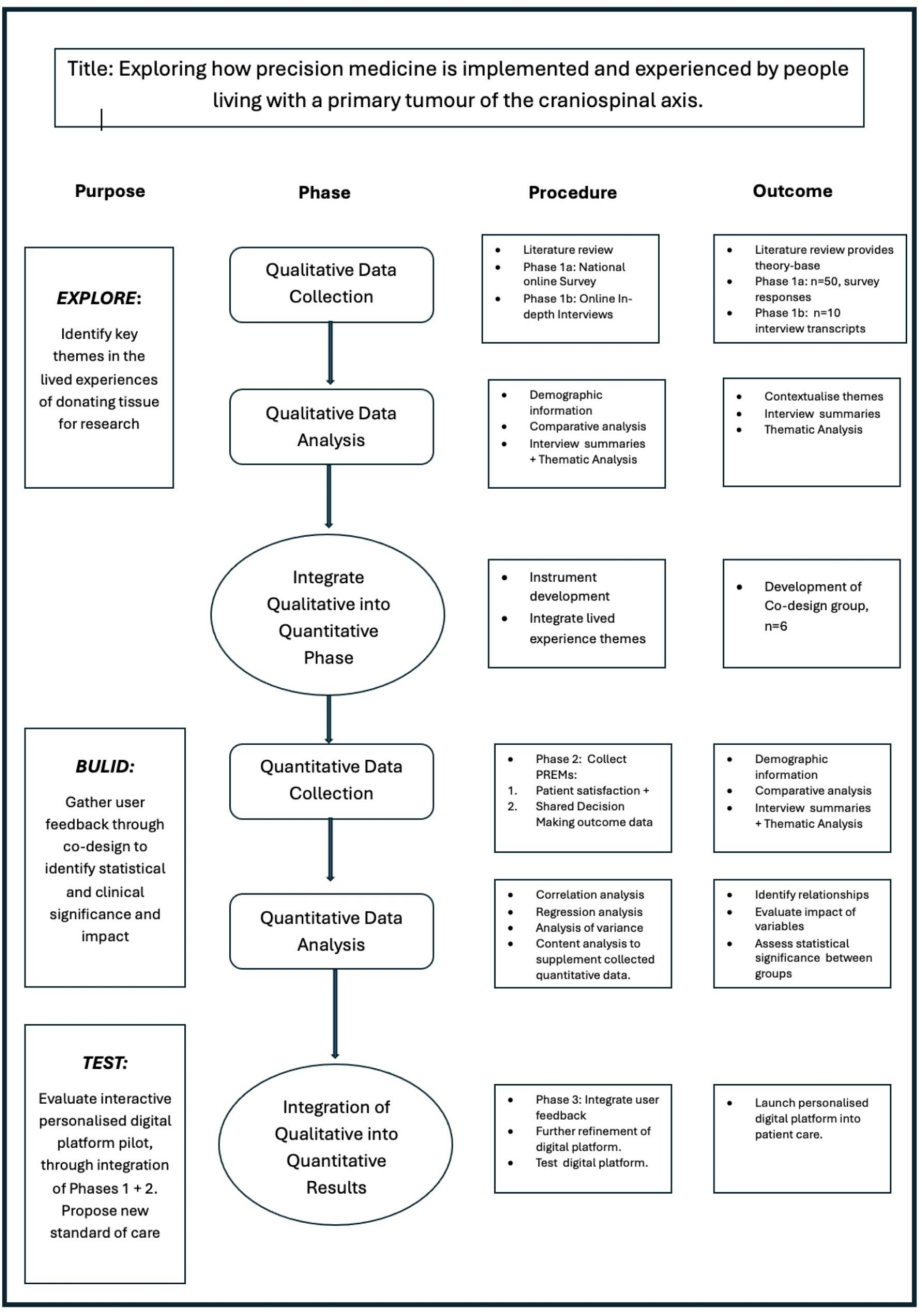
Exploratory sequential 3-phase mixed method study design

The qualitative phase delves into motivations and barriers to tissue donation, with insights informing the quantitative phase to ensure relevance and accuracy. By triangulating data, the study enhances the validity and reliability of findings. A diagrammatic overview of the general study design, produced using a mixed methods procedural chart [27] can be seen in Fig. 1.

PiCTuRE’s goal is to develop a personalised, interactive digital platform that supports informed decision-making about tissue donation and clinical trial participation. By capturing and addressing the intricacies of individual experiences, this sequential design facilitates the development of a tool that is both evidence-based and patient-centred, advancing research and clinical practices in tissue donation. The data collection process is divided into three phases: exploration, development, and testing a summary can be seen in Fig. 1.

### Scoping review

A scoping review titled “*Exploring patient perceptions and awareness of donating tissue for research within a surgical oncology setting: A scoping review”* was conducted in accordance with the Preferred Reporting Items for Systematic Reviews and Meta-Analyses for Scoping Reviews (PRISMA-ScR) guidelines [28].

A systematic search was performed across four electronic databases: CINAHL Plus, Medline, PsycINFO, and the International Bibliography of the Social Sciences. The Joanna Briggs Institute (JBI) Critical Appraisal Tools [29] were utilised to assess the quality of the primary studies; however, all relevant studies were included regardless of methodological quality. The screening process employed the Population-Concept-Context (PCC) framework to identify eligible studies.

This review aimed to investigate patient perceptions and awareness of tissue donation for research within a surgical oncology setting across diverse populations. It examined beliefs, knowledge, and attitudes, along with the broader societal factors influencing these perspectives. There is a paucity of literature within the field of neuroscience specifically addressing oncology patient cohorts and their attitudes, beliefs, and perceptions toward tissue donation for research, highlighting the need for this study.

### Phase 1: explore

Underpinned by a scoping review, Phase 1 of the PiCTuRE study will focus on understanding key themes related to the lived experiences of individuals with primary brain or spinal tumours who donate tissue for research.

The explore phase includes two stages of qualitative data collection: an online survey (Phase 1a) and semi-structured interviews (Phase 1b).

In **Phase 1a**, qualitative descriptive data will be gathered through an anonymous online survey distributed via social media and charity websites. Participants will receive study information through an embedded link in the participant information sheet before completing the survey. Consent will be obtained using a tick-box system. The survey is designed to take no longer than 15 min. A copy of the survey questions can be found in the supplementary information file.

Participants who wish to take part in **Phase 1b** will provide additional consent by sharing their email addresses for follow-up within 3 to 6 months. This stage involves online interviews conducted via Microsoft Teams with individuals who consented during Phase 1a. The interviews, lasting up to 45 min, will focus on gaining deeper insights into participants’ experiences. Consent will be documented by the researcher, and all interviews will be recorded with participant approval to support thematic analysis.

The qualitative data collected during Phase 1 will contribute to the development of a digital consent platform for tissue donation, identifying contextual factors that may influence the intervention’s outcomes [30]. The platform aims to provide personalised, tailored, and interactive support to assist patients in making informed decisions regarding tissue donation for research and participation in associated clinical trials. Thematic analysis [31, 32] will guide the refinement of the intervention and validate items for use in the subsequent mixed-method approach. Additionally, this process will provide critical insights into patients’ attitudes toward digital technology, supporting the study’s aim to enhance patient empowerment and engagement.

### Phase 2: build

Phase 2 of the PiCTuRE study focuses on the development of the digital intervention through co-design methods. Patient-reported experience data will be collected and statistically analysed to validate and refine the digital consent tool [33] Co-design and experience-based co-design approaches have proven effective in enriching the development of patient-facing interventions [34–36]. To gather feedback, two Patient Reported Experience Measures (PREMs) will be used: (1) the Client Satisfaction Questionnaire (CSQ-8), an 8-item measure to assess satisfaction with the digital platform [17, 37], and (2) a 5-item Shared Decision-Making (SDM) tool, CollaboRATE [38], which asks participants to rate the quality of their provider’s communication regarding decision making.

These tools will help capture patient perspectives on the digital intervention. Data from Phase 1a and 1b, including qualitative findings, will be integrated to further personalise and refine the digital consent tool. Participants from Phase 1b will be invited to review the online tool and provide feedback, which will be used to improve the platform. The collected satisfaction and experience data will be analysed to validate items for subsequent use, guiding the next steps in the tool’s development and ensuring that it effectively meets patient needs.

### Phase 3: test

Phase 3 of the PiCTuRE study will evaluate the newly co-designed, interactive, personalised digital consent platform, aiming to promote patient autonomy, shared decision-making, and personalised consent. Outcomes will be measured through both qualitative and quantitative methods, with a focus on user experience. Post-intervention, a survey and semi-structured interviews will be conducted to assess participant feedback and explore their lived experiences, including their views on discussing tissue donation for research with healthcare professionals. This phase will provide insights into the effectiveness of the intervention, with Patient Reported Experience Measures (PREMs) used to gather data on satisfaction and engagement [39, 40]. The qualitative data will help validate the quantitative findings and assess the overall success of the intervention [41]. Additionally, user feedback from post-intervention interviews will be analysed for statistical significance, contributing to the refinement of the intervention, and providing evidence for a potential new standard of care in cancer patient consent processes.

## Participant sampling and selection

The PiCTuRE study will be promoted across various digital platforms, including social media, charity websites, and patient support sites, using posters and e-flyers. Given the rarity of the target population, a multistage purposeful random sampling method [42] will be employed to encourage enrolment and identify individuals interested in participating in online semi-structured interviews, collaborating on the co-design of the digital platform, and providing feedback during the testing phase. Recognising the lack of inclusivity in research involving diverse populations, recruitment may also be supplemented through snowball sampling [43] via social media networks and outreach to ethnic community groups. Participants will receive the study information upon clicking the link provided in the online advertisements. An overview of the inclusion and exclusion criteria for all three phases of the study is outlined in Table 1.

**Table 1:** Overview of inclusion and exclusion criteria for phases 1 to 3

Inclusion Criteria	Exclusion Criteria
Proficiency in speaking and understanding English	Inability to communicate effectively in English
Aged 18 years or older	Under the age of 18 years old
Lived experience of having a primary tumour of the brain or spine	Unable to provide informed consent
Capable of providing informed consent	No access to internet-enabled devices (e.g., smartphone, tablet, computer)
Able to communicate via digital media	Unable to communicate using digital media
Access to an internet-enabled device (e.g., smartphone, tablet, computer)	

## Sample size

The small sample size in this study reflects the rarity of the patient cohort, influenced by factors such as the low prevalence of the tumour type, delays in presentation and diagnosis, and post-treatment survival rates.

Recruitment and sample size will be guided by the principle of data saturation [44]. Hennink and Kaiser [45] suggest that qualitative data saturation is achieved with 9–17 participants for semi-structured interviews and 4–8 participants for focus groups.

- **Phase 1a: Online Survey**– Up to 45 participants.
- **Phase 1b: Semi-structured Online Interviews**– Up to 10 participants.
- **Phase 2: Co-design Users**– Up to 6 participants.
- **Phase 3: Test Users**– Up to 6 participants.

To determine the sample size for Phase 1a, a calculation [46] was conducted using a 95% confidence level, a 5% margin of error, and an estimated population proportion of 3% within a population of 67 million. Based on these parameters, a sample size of 45 participants was deemed sufficient.

## Data storage

All electronic data will be password-protected and stored on secure university servers, while paper documentation will be kept in a locked research facility accessible only to authorised researchers. Survey data will be transferred to password-protected Excel files. Audio recordings will be deleted from devices after being securely shared with an approved transcription service. Final transcriptions will be stored as password-protected Word documents on encrypted university servers, with original audio files permanently deleted.

## Data analysis

### Phase 1a

The findings from the online cross-sectional survey will be reported in alignment with the Consensus-Based Checklist for Reporting of Survey Studies (CROSS) [44]. Thematic analysis [31] be employed to examine open-ended responses, identifying key themes and patterns. Recurring codes and themes will be subjected to comparative analysis across demographic and clinical segments, such as age groups, gender, and diagnosis. Descriptive statistics will be utilised to contextualise the themes generated through qualitative analysis. Additionally, correlation analysis will be conducted to explore relationships between variables.

### Phase 1b

Audio recordings from online interviews will be transcribed verbatim using professional transcription services to ensure accuracy and consistency. The transcriptions will then be processed in qualitative analysis software (NVivo) for detailed examination and coding. Thematic analysis [32] will be employed as the primary methodology, relying on a thorough textual analysis of the transcribed content to generate a precise and reliable dataset for subsequent analysis.

### Phase 2

PREMs outcome data will be analysed using a Statistical Package for the Social Sciences (SPSS). Correlation analysis will identify relationships between variables using correlation coefficients. Regression models will evaluate the impact of multiple variables on specific outcomes, while an Analysis of Variance (ANOVA) will assess statistically significant differences between groups. Quantitative data will be supplemented by content analysis [2, 30] of audio collected via the online co-design group sessions.

Sequential data analysis will examine the connections between findings from Phase 1 and Phase 2. These results will be triangulated to guide and inform the implementation of Phase 3.

### Phase 3

Post-intervention user experience data collected via PREMs will be analysed using the methodology outlined in phase 2 and statistically compared to the corresponding phase 2 data. Quantitative findings will be enriched by qualitative insights obtained from post-intervention online semi-structured interviews. The integration of mixed-methods data [38, 44] will facilitate a comprehensive assessment of the effectiveness of the newly developed digital tool.

## Ethics and dissemination

PICTuRE has received ethical approval from the Central University Research and Ethics Department (CUREC), University of Oxford CUREC- R79248/RE001. The protocol has been written with reference to the SPIRIT (Standard Protocol Items: Recommendations for International Trials) checklist [47] and has been registered with the International Standard Randomised Controlled Trial Number (ISRCTN) registry on the 11th November 2023, with reference number: ISRCTN12601034.

## Electronic supplementary material

Below is the link to the electronic supplementary material.

Supplementary Material 1

Supplementary Material 2

## Data Availability

No datasets were generated or analysed during the current study.

## Declarations

### Provenance and peer review

Not commissioned; peer reviewed for ethical approval prior to submission. The authors would like to acknowledge support from the Oxford Biomedical Research Council (BRC), MediServices Healthcare, Oxford Digital Health (Ox.DH) and Professor Alison Simmons, Director of the MRC (Medical Research Council) Translational Immune Discovery Unit, University of Oxford.
