# Supplementary material for: Study protocol for putting the ‘Person’ in the PiCTuRE: an exploratory sequential mixed methods-based design, exploring how precision medicine is implemented and experienced by people living with a primary tumour of the craniospinal axis
Source: BMC Cancer. 2025 Mar 7;25:420. doi: 10.1186/s12885-025-13795-9 (PMC11889933; doi:10.1186/s12885-025-13795-9)
Supplement: Supplementary file 1 — Supplementary Material 1 [file 12885_2025_13795_MOESM1_ESM.pdf]

Putting the Person in the PICTuRE: Personalised Informed Consent in Tissue donation for Research, lived Experiences.

Survey Questions

Consent to take part

1. Please note that you may only participate in this survey if you are 18 years or over.
   - I certify that I am 18 years or over [ ]   (Tick box to confirm)

2. You have read the information above and agree to participate with the understanding that the data (including any personal data) you submit will be processed accordingly, please tick the box below to start.
   - Yes, I agree to take part [ ]

Your Experience

3. Age: ....

4. What was your gender at birth
   - Male [ ]
   - Female [ ]
   - Prefer not to say [ ]

5. Which best describes your ethnicity
   - Arab [ ]
   - Asian [ ]
   - Black [ ]
   - Mixed race [ ]
   - White [ ]
   - Other [...]

6. What is / was your tumour diagnosis?
   - Spinal [ ]
   - Cranial [ ]

7. What was/is the name of your tumour?, e.g. glioma, chordoma, chondrosarcoma, other...
    - [...]

8. How long have you had your diagnosis?
    - [.............................]

9. As part of your treatment journey, were you invited to donate tissue for research?
    - Yes [ ]
    - No [ ]
    - Not sure [ ]

10. Did you find it stressful to be asked about taking part in research when faced with your tumour diagnosis?
    - Yes [ ]
    - No [ ]

11. If you found it stressful to be asked about donating tissue for research – please tell us why?
    - [...]

12. In your experience, when would have been the best time to introduce the topic of donating tissue for research?
    - In the letter inviting you to a consultation with the specialist [ ]
    - At the time of your appointment [ ]
    - After your appointment [ ]
    - On the day of surgery [ ]

13. When asked to participate in research – did you find the information easy to understand?
    - Yes [ ]
    - No [ ]

14. How was the information delivered?
    - Printed [ ]
    - Electronic [ ]

15. Did you have the opportunity to ask questions?
    - Yes [ ]
    - No [ ]

16. Who consented you to take part in research?
    - Treating clinician (Hospital) [ ]
    - Specialist Nurse [ ]
    - Research Nurse [ ]
    - Physio / OT/ Dietician / Other AHP [ ]
    - GP [ ]
    - Other [ ], [....................................]

17. Where did the consent take place?
    - Clinic room with treating clinician present [ ]
    - In a different clinic room [ ]
    - Waiting room [ ]
    - Hospital corridor [ ]
    - Hospital ward [ ]
    - Online [ ]
    - Other [ ], [............................]

18. Did you have sufficient time to consider your decision to participate or not?
    - Yes [ ]
    - No [ ]

19. Prior to agreeing to take part in research, did you have the opportunity to discuss your decision with family or friends?
    - Yes [ ]
    - No [ ]

20. If no, would you have liked the opportunity to discuss your decision with family/friends?
    - Yes [ ]
    - No [ ]

21. On a scale of 1-10, where 1 is the worst and 10 is the best, how would you rate your experience of donating tissue for research?
    - [1 2 3 4 5 6 7 8 9 10]

Digital Consent

22. Do you have experience of using digital / online portals to access your personal information e.g. online banking or online shopping?
    - Yes [ ]
    - No [ ]

23. Would you be happy to use a digital / online portal such as the NHS app to access information regarding your decision to take part in research?
    - Yes [ ]
    - No [ ]

24. The PICTuRE project will introduce the concept of " Digital Dynamic Consent". This means that consent will be obtained interactively with a participant and a clinician / researcher, using a digital portal, which can be accessed anywhere, allowing you to provide and withdraw your consent depending on the research project. The portal will aim to reduce the burden of completing multiple consent forms and excessive paperwork. Would such a portal be of interest to you?
    - Yes [ ]
    - No [ ]
25. The portal will also contain additional resources, which of the below would be of interest to you? Choose all that are relevant:
    - Active research projects [ ]
    - Completed projects [ ]
    - Frequently asked questions section [ ]
    - Information related to your primary tumour diagnosis [ ]
    - Links to tumour charities [ ]
    - Other [ ]

26. If other, please tell us what additional information you would find useful
    - [...]

27. The portal would be hosted on an NHS-grade secure online platform, which will comply with UK data protection, GDPR and confidentiality law. Would you have any concerns around security and sharing information via such a portal?
    - Yes [ ]
    - No [ ]

28. If yes, please tell us your concerns...
    - [...]

29. If no, please tell us why?
    - [...]

Interested to participate in part two of the study?

30. We kindly ask a few participants to volunteer for an additional interview. If you are maybe willing to take part in an interview, please provide your email address in the box below. If you change your mind later, there is no obligation to take part.

    A separate consent for the interview will be taken at the time.
    In further participating in PICTuRE you have the opportunity to contribute to a ground breaking national online portal for consent. Would you like to Help?
    This would involve participating in a short online interview based on your experiences of consenting to donate tissue for research.
    Your continued inputs and support are essential for the success of the project which will reduce paperwork associated with your care and the conduct of research into your condition.
    If so, please tick yes and enter your email address below
    - No, thank you [ ]
    - Yes, I would like to volunteer for an online interview [ ]

    I agree to be contacted by the researcher, my email address is

    [...]
